# Hospital and Institutional News

**Published:** 1916-07-29

**Authors:** 


### HOSPITAL AND INSTITUTIONAL NEWS.

### THE MESOPOTAMIA INQUIRY.

There is now every prospect of placing the responsibility for the breakdown of the medical arrangements of the Tigris Expedition. On Thursday last week, in both Houses of Parliament, the conduct of this campaign was the subject of debate, and it is now common knowledge that two separate inquiries are to be instituted—one in regard to the Dardanelles, and the other respecting the Mesopotamia Expedition. They are to be conducted by small bodies on which both Houses of Parliament will be represented, but are not to be confined to Parliamentarians, and the Commissioners will have power to compel the attendance of witnesses. In the House of Lords, the Earl of Wemyss, in calling attention to the breakdown of the medical arrangements in Mesopotamia, said that when people heard what went on as regarded the treatment of the wounded they rubbed their eyes and wondered whether they were back in the days of the Crimea. On the occasion of the Battle of Ctesiphon accommodation was provided for 500 men, arrangements were improvised for 2,100, and the total number of wounded was 4,500. He went on to say that the best hope of the friends of a British soldier wounded in Mesopotamia was that he would fall into the hands of the Turks. All this has happily been changed now, but it is imperative that the responsibility for the breakdown should be fixed.

### WOMEN DOCTORS FOR THE REGULAR ARMY.

It is stated by the Lobby correspondent of the *Daily News* that the War Office has asked for the services of women doctors to work in Regular Army hospitals, and that forty qualified women have been chosen for service, some of whom will be sent to Malta. This is the first occasion on which medical women have been accepted for Army service, for those serving with Red Cross units are not directly in War Office employ. The question of uniform has, it is said, not been decided; possibly the cognate question of their rank, if any, and status in general is still under consideration. The obvious course would be to appoint these ladies to commissions in the R.A.M.C.; if there is any legal or departmental difficulty about commissions for women, it should not prove impossible to abrogate it. There is a relevant consideration in the commissioned rank granted in the Canadian Army Medical Corps to nurses and matrons, who hold (and wear the badges of) rank as lieutenants and captains. If a Canadian nurse may thus hold commissioned rank, it would be invidious to deny it also to British women doctors, under whom the former might find themselves serving.

### RECOGNITION FOR THE WOUNDED.

The movement in favour of bestowing badges upon men who have been wounded or disabled is making headway, but though France was apparently before us in this matter, the French example is being modified in various ways. A distinction is made in the badges given respectively to the sick and to the wounded. The wounded, still capable of some form of active service, are to receive a gold stripe, while those who have been forced by disablement to return to civil life are to receive a silver medallion. The difficulty is to give everyone entitled to recognition a badge which yet shall not seem everybody's property, while at the same time avoiding a series of decorations which may seem to make invidious distinctions. Incapacity presumably includes the victims of disease, who may not have suffered the scars of or loss of limb which has been so often in the past the sole distinction accorded to the hero.

### BILL FOR WAR CHARITIES.

Legislation will follow quickly upon the Report of the Committee on War Charities, and a Bill providing for the registration of funds and registration of street collections will shortly be introduced by the Home Secretary. Presumably the Bill will follow closely the recommendations of the Committee, the details of which are now familiar to the public, the main trend being that registration should only be granted where at least three approved persons constitute the responsible committee, where accounts are properly kept and audited and open to the inspection of a public authority, and where there is a separate banking account for all the money subscribed. With the need for such registration and the general findings of the Committee we dealt in the leading article of our issue of July 22. The weak point of the Report has generally been conceded to lie in the large number of individual authorities from which registration could be obtained; to counteract this applications will need to be fully advertised in the public press, and we hope that provision will be made for this very necessary safeguard against overlapping and fraud. But how will organisations stand which are at the same time long established, and yet in some aspects war charities, such as voluntary hospitals providing beds for the wounded?

### THE HOTEL AS A HOSPITAL.

In their demand for buildings which might be converted into military hospitals the authorities have directed their attention almost wholly to public offices and institutions. Schools, municipal halls and buildings, picture galleries, have in turn been raided, not always with due regard to their fitness for hospital purposes, as we have had frequent occasion to show. Schools in particular are frequently unfitted for hospital use, and much money has to be spent in adapting them. Thus the policy of economy which, in the widest sense, doubtless dictated this policy has often defeated its own end, and a suggestion has now been made that private buildings should by preference be appropriated. The first type of building which suggests itself as promising is the hotel, and since more than one has been taken over for Government purposes in London, why should they not be taken over elsewhere? A big hotel generally has grounds, kitchens, lavatory accommodation, lifts, and general service arrangements ready to hand. Apart from the ground floor it does not usually have rooms equal in size to the usual big military ward. But that does not seem an insuperable difficulty. Why, then, should not the hotel be commandeered and the public services not incommoded?

### ELECTRIC FANS FOR HOSPITALS.

Is there a shortage of electric fans at the hospitals in the Persian Gulf and in Egypt? This is a very important question, seeing that the temperature in the Persian Gulf rises to 120 degrees with a still, steaming heat, and that Alexandria and other places in Egypt are subject to great heat. A correspondent writing to the *Electrical Review* relates that a lady connected with the nursing profession, and having an intimate knowledge of the East, informed him that the supply of fans in the military hospitals is totally inadequate, and he makes the very useful suggestion that as the weather in this country is more suited for radiators than fans, there must be many fans in Great Britain belonging to firms in the electrical trade who would be glad to give these to relieve the discomfort of our brave men in tropical climates. Before anything is done, however, he suggests that information should be obtained regarding the different electricity pressures at hospitals in these tropical climates, since it is obviously no use sending a hospital supplied with 100-volt continuous current a 200-volt alternating-current induction fan. There should surely be some means of obtaining information of this kind without delay, and it is to be hoped that this will be done so that this correspondent's suggestion may be carried into effect with all possible speed.

### THE EFFECT OF THE WAR UPON RINGWORM.

At first sight there would not seem to be any connection between the war on the Continent and the spread of ringworm among English school-children. That such a connection does exist, however, is shown by the report to the Salop Education Committee of the school medical officer, in which it is stated that, owing to the number of wounded soldiers requiring $x$-ray treatment at the Royal Salop Infirmary, it is impossible for cases of ringworm to be dealt with there; and that no other installation is available in the county for the $x$-ray treatment of these cases among school-children. The report also states that $x$-rays constitute the "only known method of cure." It is doubtless the fact that $x$-rays are the most reliable and quickest method of treatment, but it seems a pity for a medical officer to give a lay committee the impression that no other treatment is known. If that were in fact the case, clearly no case of ringworm would ever have been cured prior to the application of $x$-rays for this purpose some ten or twelve years ago. It was resolved, in consequence of the report, that such children suffering from ringworm as the school medical officer should select should be admitted to school, although they might be suffering from this parasitic disease.

### A SECOND RAID ON THE SCHOOLS.

The Cardiff Education Committee has decided to place a further group of schools at the disposal of the military authorities for hospital purposes as required. This is a very serious step, and may involve considerable educational disorganisation; and connoisseurs in irony have not failed to note that this decision comes at the very moment when the need for increased educational effort is being emphasised on all hands. May we, then, suppose that the first step in educational reform is to close our present schools? If the Government had a policy, that would be the deduction; but hospital men are aware that the military authorities have, doubtless under advice, shown an ineradicable preference for adopting public buildings, whatever their nature, and largely irrespective of their suitability. An alternative plan is discussed in our Note on "Hotels as Hospitals," which points the way to a more statesmanlike system. The public Services should not be made to prey upon each other, and a school is usually a bad hospital. We commend to Cardiff the example of Leicester in making a fight for its schools and for the wounded.

### SOLDIERS' EMBROIDERY.

The movement in favour of teaching handicrafts to disabled soldiers has led to a series of exhibitions of their work. Four have been held at Newcastle, the latest of which took place last week. It was remarkable for the ingenious and accomplished examples of embroidery which it contained. We understand that some of the exhibits have been made by men who have since returned to active service, during which apparently they still find time to carry on the handicrafts learnt by them at Newcastle. A great point is made by the Northumberland Handicrafts Guild of the value of these handicrafts in teaching permanently disabled men to earn a livelihood or to supplement reduced earnings. That is all very well, but we must again point out that the responsibility for providing the permanently disabled soldier with a livelihood rests not with the unfortunate man but with the Pensions authority. This, so slow and grudging in its action, has only been empowered to distribute an additional £6,000,000 by the pressure of public opinion; and there is a distinct danger that it may seek to evade its duty by "taking into consideration" any earnings which a disabled man has been taught to make by some freak activity or other. That is the danger attending an attempt to make, let us say, a one-handed soldier "self-supporting" by teaching him embroidery.

### THE LIMITS OF DISPENSARY USEFULNESS.

A conjoint scheme has been approved by the Shoreditch and Islington Borough Councils for an agreement with the Royal Hospital for Diseases of the Chest, City Road, whereby a tuberculosis dispensary is to be provided for insured and uninsured persons who are resident in the districts. The dispensary will be situated at the hospital. While the establishment of these dispensaries is to be welcomed, unfortunately the fact remains that the campaign against consumption will continue to be half-hearted so long as no adequate provision is made for advanced cases in sanatoria, and for the institutional treatment of other patients in accordance with their medical requirements and not within fixed limits of time. As it is, an insured person suffering from tuberculosis is lucky if he receives sanatorium treatment at all, and in any case the chance that he will be discharged at the end of three months is a very great one. Such improvement as may be thus effected is almost bound to be temporary, and amounts to little more than a tinkering with the case. So long as these half-hearted measures prevail dispensaries can do little beyond recording the prevalence of the disease, and the failure of existing half-hearted attempts to deal with it.

### AFFORESTATION WORK FOR DISCHARGED SANATORIUM PATIENTS.

The attention of medical practitioners and approved societies has been directed by the Glasgow Insurance Committee to a report prepared by their clerk, Mr. William Jones, on the committee's experience in the treatment of insured persons suffering from phthisis in sanatoria and hospitals and the "after history" of these patients. The conclusion arrived at is that sanatorium treatment does effect an improvement in the health of a large proportion of the patients; but that, except in very early cases, the improvement is of a somewhat temporary character. The immediate return of the sanatorium patient to normal conditions of living is not attended with any reasonable measure of success, and further provision should be made before a patient is allowed to do so. The farm colony or similar institution seems, to the Glasgow Insurance Committee, most likely to secure the objects in view. The committee has resolved to get into touch with those in charge of the national afforestation scheme, with a view to arranging that patients discharged from sanatoria or hospitals should be afforded opportunities of employment.

### INFANTILE PARALYSIS AT ABERDEEN.

A distinctly serious outbreak of poliomyelitis (infantile paralysis) is reported from Aberdeen by the medical officer of health for that city. The last case before this epidemic was a single one, notified in July 1915. About the middle of June 1916 nine cases were reported by a single practitioner, and the medical officer of health at once warned the Aberdeen doctors by circular; within the month twenty-nine cases in all were notified, and ten more in the first week of July. The cases are practically confined to the working classes; but most of them have occurred in families which are well cared for, not overcrowded, and above the average in cleanliness. In only one family have two indisputable cases occurred, though in another one there is one certain case and one doubtful. The infected households are mostly in different streets, and fairly widely separated. The majority of the families are quite unknown to one another; neither school nor milk appears to have any traceable influence on distribution. All the children, except three, are under three years of age. Only two cases have proved fatal, and most of the paralyses are of mild type. Endeavours to isolate a micro-organism are being prosecuted, but so far no success has been reported.

### A PROPOSED SEMI-CONVALESCENT HOME.

An important extension has been decided on in connection with the Liverpool Children's Infirmary. It is nine years since the present infirmary was built, but the 110 beds and the out-patient department have proved insufficient to keep pace with the waiting list. An appeal, therefore, has been issued for £20,000, so as to form an "auxiliary establishment" of fifty beds. Since building operations might not be practicable at the present time, the committee propose in the first instance to adapt a large house and garden which is understood to be available not far from the present buildings. The idea is that this house not only could be adapted, but that the site would allow of extensions when the time and opportunity came for making them. The suggested proximity of the new building is expected to enable the present honorary medical staff to supervise the work. The idea is to send semi-convalescent cases to the new building, and thereby to release beds in the infirmary for fresh cases.

### THE THREEPENNY DOCTOR CONVICTED.

The trial of Henry Jelley for causing the death of a woman by performing an illegal operation upon her has ended in a conviction for manslaughter and a sentence of three years' penal servitude. Thus ends for the present the career of the Homerton "threepenny doctor," a ruffian who is a disgrace to the medical profession, into which he should never have been admitted. The examining corporation which gave him his qualifying diploma, and the examiners who "passed" him, have a heavy responsibility to bear, and should be brought to book for it. Not because Jelley has procured abortion, for neither the Society of Apothecaries nor any other body can eliminate potential criminals, but because he was never at any time a fit and proper person by training or knowledge to be entrusted with the lives or health of human beings. On his own statement Jelley obtained his qualification of L.M.S.S.A. after six weeks' study. It is not merely the facts of the present case, and the amazing statements of the prisoner in his defence, but the whole history of this man's career since he obtained registration that condemn him. The "nursing home" which he conducted at Homerton was a public scandal: we say this with assurance, because two or three years ago a representative of this Journal went to inspect it, and his report was so strongly worded that in the present unsatisfactory state of the law of libel it was impossible to publish it. We fear the sentence passed upon him is unlikely to deter Jelley from returning to his evil courses on his release.

### THE REFORM OF THE FUNERAL.

To the proposals on this subject recently mentioned in these Notes must be added another made by Dr. Barnes, the acting medical officer of health for Southport, which seems to show that the subject is engaging considerable attention in the North of England. In a paper read before the British Embalmers' Society and the British Undertakers' Association Dr. Barnes made two principal proposals as to burial from the sanitary point of view. The first was that the medical officer of health alone should be able to give permission for the body of a person who had died of an infectious disease to remain for longer than forty-eight hours in a private house. The second was that the massive coffins which custom had sanctioned in the illogical attempt at once to preserve and dispose of the body should be done away with, and in the interests of sanitation some such material as wicker or papier mâché should be substituted. He pointed out that the Burial Service rather studiously avoided the mention of coffins, and that, though a parishioner had a right to be buried in his own churchyard, where was the rule that gave him a right to have a large chest buried with him also? We believe that the slow but steady growth in the popularity of cremation is better worth medical support than any modification of wooden coffins. The classic honours of the pyre are more likely to convert sentiment than the sanitary advantages of papier mâché.

### THE MOTHERS' UNION.

This organisation, which was established in 1887, is appealing for the sum of £50,000 required for the erection of a proposed central building, to be called (after the foundress of the Union) "the Mary Sumner House." What site is it to occupy? Since building operations cannot be begun until after the war, it is contemplated that temporary premises, which are immediately available, shall be taken. The completed Institute is to contain a central hall, large enough for council meetings and lectures, and a maternity centre worked by experts. The Institute is also to become a training centre for social and Mothers' Union workers at home and overseas. The scheme has the commendation of the Archbishops of Canterbury and York, and the appeal is issued by the Central President, Mothers' Union Offices, Church House, Westminster. The honorary treasurers of the special fund are Lord Parmoor, K.C.V.O., and the Hon. Evelyn Hubbard; contributions may be sent to them, or to the Central President, at the offices.

### HOSPITALS AND THE AUGUST HOLIDAYS.

It is not only munition workers who have been asked, or have volunteered, to forgo their annual August holiday. A similar decision is affecting some of the war hospital supply depôts, since it is felt that the stream of hospital supplies must be kept as high as that of munitions. Possibly in this department of hospital work the practice may spread, since, if one class of the community is asked to forgo their holiday, it is desirable that they should feel that the burden, a very real one to munition workers, does not fall on them alone. The committee of an Irish war hospital supply depôt has been prompt to apply the Prime Minister's appeal to its own workers.

### AN EVENTFUL YEAR.

Few hospitals can have had a more eventful year than the one which the East Suffolk and Ipswich Hospital has experienced. Having previously raised the number of beds from 143 to 260, during the past year a new temporary hospital block of 120 beds has been built and occupied. One result of this has been that the expenditure has more than doubled, rising, to give the figures, from £11,400 to £23,300. But not only has the number of in-patients increased by nearly 1,000, but this number refused to average itself out, so that at one time there were 377 patients in the hospital and at another only 166, a fluctuation due, no doubt, to the periodic lulls or rushes in the arrival of convoys. Luckily the income showed a substantial increase, but on revaluation the hospital's securities were seen to have decreased by practically £6,000; but, with the help of the Government grant, the deficit on the year is not quite £1,000. At the meeting last week Mr. J. D. Cobbold, the chairman, stated that the War Office had lately asked them to provide another sixty beds. At the present time there were 290 wounded soldiers in the hospital.

### A BATTLE BED AT GRIMSBY.

It has been decided to endow a John Travers Cornwell cot in the Grimsby Hospital in memory of Cornwell, the boy hero of the Battle of Jutland, who died in one of the wards. This decision has been come to by the District Hospital Committee, and whether or not a national tribute be added to it, a Cornwell cot should certainly be placed in the institution. Indeed, we regret that, so far as memorials of this character are concerned, hospitals have not already more to show than they have, considering the part they have played in the war and the number of wounded which has passed through them. Possibly the future may see a larger number of endowed beds associated with the war. It must be hoped so, since the defect of the hospitals at the present time is, as it has so long been, an increased list of waiting cases. Since, with the modest help of the War Office, the net total number of beds has been so frequently raised by hospitals which have added wards for the reception of the wounded, the aim must be, when peace returns, to maintain, if possible, the higher number for the benefit of the civilian population. As a means to this end, and even apart from the particular heroisms which it is desired to commemorate, every endowed bed associated with the war is much to be welcomed.

### A NEW MATERNITY REST HOME.

The new Maternity Rest Home which has been opened at Liverpool in connection with the Maternity Hospital claims to be a pioneer institution. Its object is to provide rest and treatment for expectant mothers suffering from illness in the course of their pregnancy. The president of the Rest Home, Mr. John Rankin, has described it as the first and best equipped home of its kind in England, the establishment of which has been due to the work of Mrs. Tom Temple, assisted by Dr. Hope, medical officer of health. The opening of the Home, in fact, is the result of a persistent agitation for the devotion of hospital beds to its particular purpose, which adds thereby an important institutional branch to the ante-natal work of the Maternity Hospital.

### THE ROD AS A SOURCE OF INCOME.

The Coventry and Warwickshire Hospital is to benefit from a new source of income, which the Earl of Craven has placed at its disposal. On Lord Craven's initiative a charge of half a guinea is to be made for fishing at Coombe, which fee entitles the fisherman to two rods. The whole of the receipts is to be given to the hospital, and arrangements have been made for the issue of tickets at the Abbey. Mr. W. J. Wormwell, the chairman, hopes that the hospital may benefit by the plan, which may be commended to landlords in other districts. Anglers who remain in civil life, of all sportsmen, will never be deterred by the turmoil of the world from their favourite sport, whose appeal is more rooted in a simple love of Nature than either hunting or shooting. It will be interesting to note the financial result of the experiment.

### THE BIRMINGHAM ROYAL INSTITUTION FOR THE BLIND.

Two things were strikingly brought out at the annual meeting of the Birmingham Royal Institution for the Blind, Edgbaston, and both were the subject of comment by the chairman of the general committee, Mr. Alfred Wilson. One was the beneficial results which have followed the compulsory notification of infantile ophthalmia, and the other the financial success which may be accomplished by a trading department for the blind when run on sound, business lines. With regard to infantile ophthalmia, which was formerly responsible for nearly 40 per cent. of the blindness among children in Greater Birmingham, since notification was made compulsory, 1,150 cases have occurred, and out of this number total blindness followed in four cases only. Although the trading department showed a record turnover, the general finances of the institution are not in such a flourishing state; for there is a deficit of £1,763. It was mentioned that the pupils are now being taught in mixed classes, which was found greatly to facilitate the organisation of the school.

### INSTITUTIONAL PORTERS AND WAR SERVICE.

Different Boards of Guardians hold different views on the question as to whether workhouse porters can be said to be engaged in work of national importance. The Ashby Tribunal recently granted exemption to the workhouse porter, but an appeal has been entered by the military authorities against the decision on the grounds that the man is fit for general military service, that he is not doing work of national importance, and that he may easily be replaced by someone over military age or a discharged soldier. The Guardians, however, have decided to fight the appeal, and have obtained legal assistance to that end. The porter at the Southwell Workhouse, who had already served in the Sherwood Foresters for a year, has again been called up; and there was actually a division of opinion among the Guardians whether the Board should contribute a few shillings weekly and bring up the porter's pay to the amount of his civilian wages. Eventually the momentous matter was adjourned.

### THE M.A.B. FOOD BILL.

The experience of the Metropolitan Asylums Board in respect of the purchase of supplies during 1915, as disclosed by the recently published annual report, throws considerable light upon the effect of the huge increases in price during that year as regards contract supplies for institutional use. The gross total value of all the supplies arranged for centrally, under formal contracts and otherwise, was about £580,000, and of this sum rather over £95,000 referred to certain minor contracts of which no reliable estimate of value can be given, and small purchases of non-contract goods at the various institutions. Of the contracts tabulated, and used to discover increases in percentage cost, 186 were for the chief articles of food, 54 for coal and coke, and 22 for soap, soda, and the like; these contracts were for so short a period, in some instances, as one week, a large number were fortnightly and monthly; therefore prices can be considered generally as current. Of the leading commodities at the end of 1915 as compared with their cost two years before the following illustrations of increases may be quoted: Fish 175 per cent., bread 74 per cent., beef about 69 per cent., and coal 34 per cent. While butter has risen 25 per cent., margarine has advanced 12 per cent. Milk, which is 25 per cent. higher, is, as delivered at the institutions, tested by the Gerber apparatus, so only a small number of samples are sent for professional analysis, but of 17 of these it is noted that 11 were satisfactory and 6 unsatisfactory.

### A REMARKABLE LETTER.

In publishing the following extracts from a letter of thanks received at one of our large hospitals, it should perhaps be explained that to us this interest does not chiefly lie in the quaint spelling. In a language like English, which has scorned phonetics, all spelling is quaint, and whether any particular arrangement of letters be correct or incorrect becomes, alas! a purely academic matter. What is not academic is the liveliness of the letter, which in turn reminds us how little education, as commonly understood, has to do with style, and how personal a gift is literary ability. "i must tell you" (the writer remarks) "that i am getting on very well indeed and that people would not think that i had 2 fractured thoyes if they seen me walking—along the levill ground but i must tell you that i am a very Bad Staire Climer at present but i hope to get a lot better on that as time goes on as i am as sound as a bell of brass in side my Body. When the wind is very rugh and cold it catches my ledgs and makes them stiff and causes them to pain me a little then i was counting up all my axidents one day and it run up to about 30 little ones and big ones and i thought it was not bad for a boy of my adge i thought please convey my Best wishes to all the nurses and Sisters and doctors and to all the staff and i hope you wont forget my Sister—as was in charge of the ward as i was in at your infirmery. And if there is a letter laying at your office for me i should be extreamiley ablidged to you if you would forward it on to me at once i remain yours very Sincerely ——." The writer of this letter was sixty-four years of age. In regretting not being able to send a subscription, he explains that "the price of living is gone up so much since the war started that i find it impossible to save a penney now out of my—— money to help aney good Cause if i wanted to but i must not complane as a little is Better than non." Few educated people could write such a lively letter of thanks, or remain cheerful in such stress of illness and circumstances.

### DUTY-FREE SPIRIT FOR HOSPITALS.

It is really very difficult to understand what causes the delay in settling the question as to the conditions under which hospitals shall receive a rebate of the duty on spirit used for medicinal purposes. When we consider that had the Government been allowed to carry out its original intention in this matter, hospitals would have been enjoying the use of duty-free alcohol for many months, and would have been thousands of pounds in pocket, it is very annoying to know that the whole business is still hung up in the committee room as it were. Last week, in the House of Commons, Sir Philip Magnus reminded the Chancellor of the Exchequer that hospital authorities are still waiting to know what the promised relief is going to be, and when they are going to get it. He asked whether the Committee which was appointed to consider the question had yet reported and, if so, when he proposed to take action on the decision at which the Committee had arrived. Mr. McKenna's reply was to the effect that the Committee had reported, that the Report was now under consideration, and that he hoped to be able to come to an immediate decision on the recommendations. It is to be hoped that when effect is given to this decision it will be found that the concession has been worth waiting for.

### THE SEAMEN'S HOSPITAL SOCIETY.

The annual report of this Society, which administers not only the well-known *Dreadnought* Hospital, Greenwich, but also the Albert Dock Hospital, two dispensaries, and two medical schools, bears upon its cover the portrait of a smiling member of the Naval Division who has lost a leg in the service of the country. One very interesting page in the statistical part contains the usual tables respecting the cost of the laundry. It seems that during the year 572,884 pieces were washed on the premises of the two hospitals, at a total cost, including interest on capital, depreciation on buildings and machinery, of £1,470, or rather more than a halfpenny each. This, doubtless, is rather more than the work could have been done for by contract; but the Society has the satisfaction of knowing that disinfection and suchlike necessary processes are carried out under close supervision, and therefore without any chance of slipshod make-believe.

### THIS WEEK'S DRUG MARKET.

Since the last report was written there has been a remarkable fall in the price of bromides, the value of potassium bromide being more than halved and the ammonium and sodium salts having been reduced almost in the same proportion. The market had been unsettled for some little time, but such a sharp drop in prices as that which has actually taken place was quite unexpected and entails a considerable loss to some holders. This event has imparted a nervous feeling to operators in other drugs, and buyers are confining their purchases to their immediate requirements. Sodium salicylate and salicylic acid are again rather lower in price, and there is a lower tendency in the prices of acetylsalicylic acid, acetanilide, sulphonal, and hexamine; in fact, the value of few of the synthetic drugs is so firmly maintained as it was, one of the few exceptions being phenacetin, which is difficult to obtain. Citric acid, cream of tartar, and tartaric acid also have a lower price tendency. Camphor is one of the few drugs with an upward price tendency.

### TO OUR READERS.

**Contributions are specially invited from any of our readers to these columns. They should deal with topical subjects and news. They must be authenticated for the information of the Editor only. The minimum payment if published is 5s. There is no hard-and-fast rule as to space, but notes of about twenty lines in length are preferred.**

