# Erratum

**Published:** 1916-09-02

**Authors:** 


### Erratum.

IN our issue for July 29 (p. 402) a remark to the effect that *x*-rays constitute the only method of cure for ringworm was attributed to the school medical officer of the Salop Education Committee (Dr. James Wheatley). We now learn that the statement was not his, but that of another doctor who is a member of that committee. We therefore apologise to Dr. Wheatley for attributing to him a statement which he did not make, and trust that the mistake has not caused him any inconvenience.

